# Diagnostic Delays in Parkinson’s Disease in Thailand: Clinical Pitfalls and Health System Barriers

**DOI:** 10.3390/life15101513

**Published:** 2025-09-25

**Authors:** Praween Lolekha, Piriya Jieamanukulkit

**Affiliations:** Neurology Division, Department of Internal Medicine, Faculty of Medicine, Thammasat University, Pathumthani 12120, Thailand; piriyajieama@gmail.com

**Keywords:** Parkinson’s disease, diagnosis delay, aging and brain health, health disparities, Thailand

## Abstract

Background: Parkinson’s disease (PD) is a progressive neurodegenerative disorder in which early diagnosis improves quality of life and reduces disability. However, diagnostic delays remain common, particularly in low- and middle-income countries. This study investigated clinical and system-level factors contributing to diagnostic delay in Thailand. Methods: A retrospective chart review was conducted on patients newly diagnosed with PD at Thammasat University Hospital between June 2020 and June 2024. Demographic, clinical, and healthcare access data were analyzed. Diagnostic intervals were defined as onset-to-visit (OTV), visit-to-diagnosis (VTD), and onset-to-diagnosis (OTD). Age-at-onset groups included early-onset Parkinson’s disease (EOPD, <50 years), regular-onset PD, and very-late-onset PD (≥80 years). Results: Of 1093 patients screened, 109 newly diagnosed PD cases met the inclusion criteria. The median OTV was 360 days, and the median VTD was 10 days. Tremor was the most frequent initial symptom (75%). Patients with higher education and extended family support sought care earlier, whereas those under the Universal Coverage Scheme (UCS) experienced longer OTD durations (median, 541 vs. 181 days in privately insured patients). More than half of patients were initially misdiagnosed, especially when first evaluated by non-neurologists. Conclusions: Diagnostic delay in Thai PD patients stems mainly from late help-seeking and inequities in healthcare access. Addressing these gaps requires public awareness, physician training, streamlined UCS referral pathways, and adoption of biomarker-supported digital tools to ensure earlier and more equitable diagnosis.

## 1. Introduction

Parkinson’s disease (PD), a progressive neurodegenerative disorder that predominantly affects older adults, poses a growing challenge to brain health equity worldwide. In the context of an aging global population, delayed diagnosis of PD can compromise long-term neurological health, diminish quality of life, and exacerbate healthcare disparities, particularly in low- and middle-income countries (LMICs). Such delays postpone the initiation of effective therapies that could alleviate motor symptoms and preserve functional independence. They also contribute to increased disease burden, accelerated disability, and higher healthcare costs associated with complications and the need for more intensive care services [1]. These consequences disproportionately impact vulnerable populations and highlight the urgency of fostering equitable, accessible, and inclusive neurological care.

In Thailand, as in many Asian countries, the aging population has made PD an increasingly urgent public health concern. Despite this, healthcare access is often delayed until patients develop more advanced or disruptive symptoms, largely due to low disease awareness, under-recognition of early symptoms, and systemic healthcare barriers [2,3]. Variability in healthcare accessibility, limited availability of specialists, and disparities in public knowledge further contribute to delayed recognition and diagnosis in the Thai context. 

To address these challenges, the present study aimed to investigate factors contributing to diagnostic delays in Thai patients with PD and to identify common diagnostic pitfalls that hinder timely recognition and intervention.

## 2. Materials and Methods

A retrospective chart review was conducted on patients newly diagnosed with PD (ICD-10 code G20) at Thammasat University Hospital, a tertiary care center located on the northern outskirts of Bangkok, between June 2020 and June 2024. A probable diagnosis of PD was established by neurologists, movement disorder specialists, or referral physicians, and the Movement Disorder Society Parkinson’s Disease (MDS-PD) diagnostic criteria [4] were retrospectively applied to confirm diagnostic consistency. Patients were excluded if they had received a PD diagnosis prior to the study period or if their clinical symptoms and signs, upon review, were inconsistent with PD. The minimum required sample size of 78 patients was estimated based on a previously published study [5] using the formula: n = [(Z^2^_a_/_2_ × σ^2^)/d^2^], assuming a 95% confidence level and acceptable margin of error.

Demographic and clinical data were extracted from medical records, including age at motor symptom onset, symptom duration, time to diagnosis, sex, residence, healthcare coverage, type of first physician consulted, presenting motor and non-motor symptoms, predominant motor features and side of onset, Hoehn and Yahr (HY) staging at diagnosis, use of neuroimaging, and initial treatment following diagnosis. Diagnostic intervals were defined as onset-to-visit (OTV), visit-to-diagnosis (VTD), and onset-to-diagnosis (OTD). Data extraction was independently performed by a trained reviewer and cross-checked by a movement disorder specialist to enhance reliability. Variables with substantial missing data were excluded from analyses. Continuous variables were reported as the mean ± standard deviation (SD) or median with interquartile range (IQR), as appropriate. Categorical variables were summarized as frequencies and percentages. Comparisons of continuous variables were performed using an independent *t*-test or Mann–Whitney U test, while categorical variables were compared using a chi-squared test or Fisher’s Exact test. All statistical tests were two-tailed, with a significance level set at *p* < 0.05. 

This study was approved by the Human Research Ethics Committee of Thammasat University (MTU-EC-IM-0-203/67) and registered with the Thai Clinical Trials Registry (TCTR20250709001).

## 3. Results

**Figure 1:**
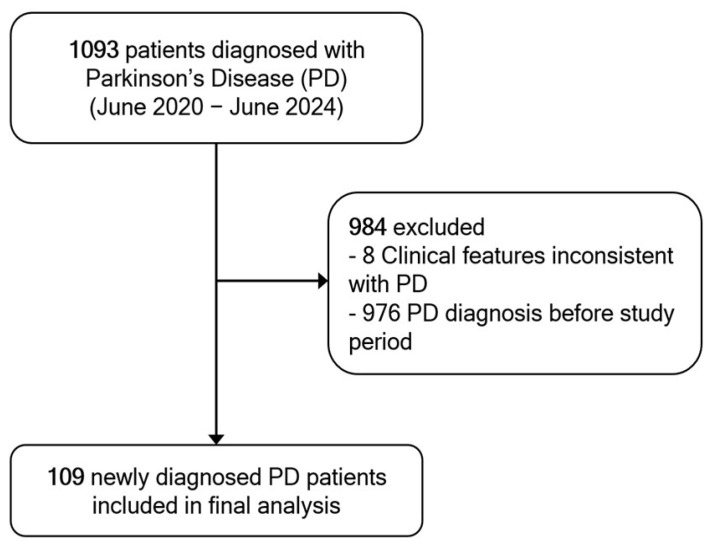
Flow diagram of patient selection. A total of 1093 patients diagnosed with Parkinson’s disease (PD) were screened at Thammasat University Hospital between June 2020 and June 2024. After excluding those with prior PD diagnosis or inconsistent features, 109 newly diagnosed PD cases were included in the final analysis.

A total of 1093 patients were diagnosed with PD during the study period. Of these, 976 were excluded due to a prior PD diagnosis before the study period, and 8 patients were excluded because their clinical features were inconsistent with a probable PD diagnosis upon review. Consequently, 109 newly diagnosed patients met the inclusion criteria and were included in the final analysis (Figure 1). 

The cohort exhibited a slight male predominance (59.63%). The mean age at motor symptom onset was 69.13 ± 9.29 years. Most patients (62.38%) were covered by the Civil Servant Medical Benefit Scheme (CSMBS), followed by self-pay/private insurance (16.51%) and the Universal Coverage Scheme (UCS) (15.59%). A family history of PD was documented in 46.79% of cases; 11.76% confirmed as positive. Approximately 75.23% of patients resided within 80 km of the hospital. Regarding initial healthcare access, 62.39% of patients were first assessed by non-neurologists in outpatient clinics, whereas 37.61% were directly evaluated by neurologists (Table 1). 

Movement disorder specialists confirmed the diagnosis in 55.05% of cases, followed by general neurologists (39.45%), internal medicine physicians (4.59%), and general practitioners (0.92%). Because the time intervals exhibited high variability and a skewed distribution, the median was chosen as the most appropriate measure of central tendency. The median OTV duration was 360 days (IQR 150–720), while the median VTD duration was 10 days (IQR 1–30). The median VTD and OTD durations were significantly shorter in patients who were initially assessed by neurologists, whereas the median OTV duration did not differ significantly between groups (Table 2).

**Figure 2:**
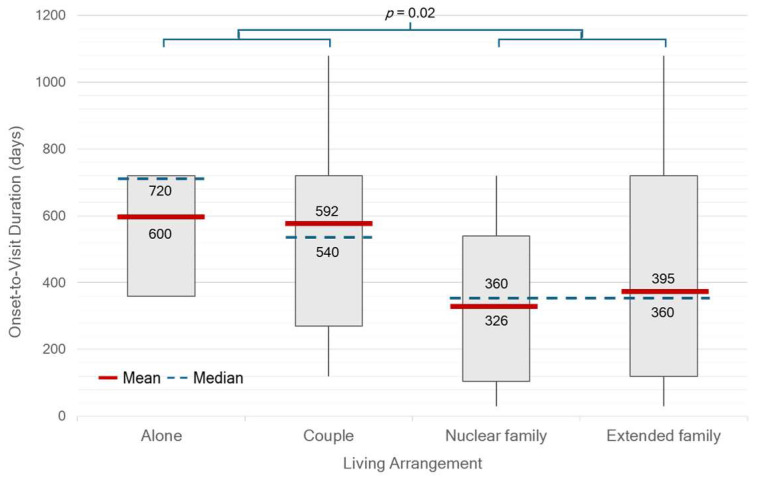
Onset-to-visit (OTV) duration by living arrangement among 109 Thai patients with newly diagnosed Parkinson’s disease. Red lines indicate mean values, blue dashed lines indicate medians, and numbers indicate mean/median. Patients living alone or only with a partner experienced significantly longer OTV duration compared with those in family households (*p* = 0.02).

**Figure 3:**
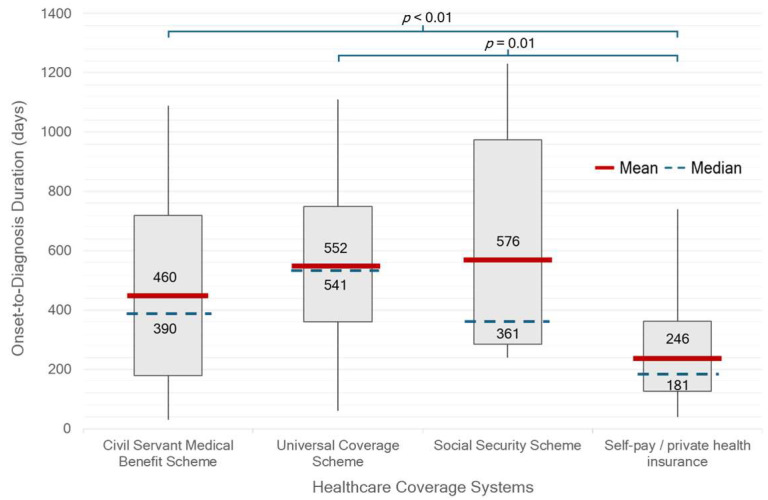
Onset-to-diagnosis (OTD) duration (days) by healthcare coverage system among 109 Thai patients with newly diagnosed Parkinson’s disease. Red lines indicate mean values, blue dashed lines indicate medians, and numbers indicate mean/median. Patients with self-pay/private insurance had significantly shorter OTD duration than those under the Universal Coverage Scheme (UCS; *p* = 0.01) and the Civil Servant Medical Benefit Scheme (CSMBS; *p* < 0.01).

**Figure 4:**
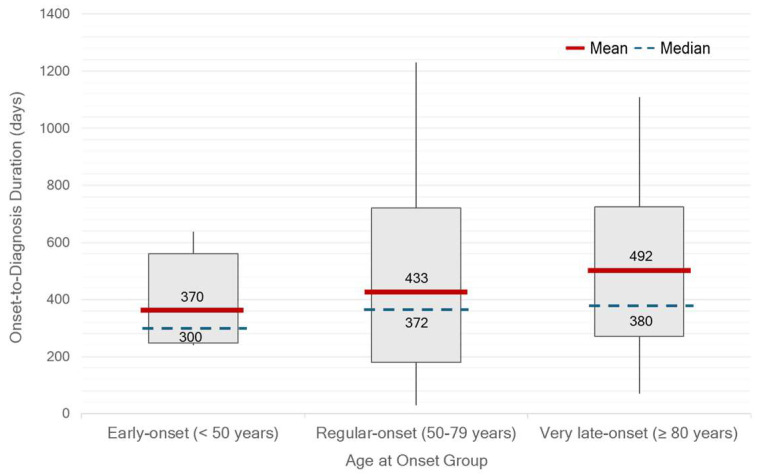
Onset-to-diagnosis (OTD) duration (days) by age-at-onset group in Thai patients with Parkinson’s disease (n = 109). Red lines indicate mean values, blue dashed lines indicate medians, and numbers indicate mean/median. No statistically significant differences were observed among groups, although patients with very-late-onset (≥80 years) tended to have longer OTD durations.

There were significant differences in median OTV duration between single- or couple-households and family households (*p* = 0.02) (Figure 2). Patients who were self-paying or covered by private insurance had a significantly shorter median OTD duration of 181 days (IQR 127–362) compared with those under other healthcare schemes (390 days, IQR 241–721; *p* < 0.01). In contrast, patients receiving care under the UCS had the longest median duration of 541 days (IQR 361–750) (Figure 3). There was no statistically significant difference in median OTD duration between early-onset Parkinson’s disease (EOPD, <50 years), regular-onset PD (50–79 years), and very-late-onset PD (≥80 years). However, diagnostic delays appeared longer in patients with very-late-onset PD (Figure 4). No significant differences in OTV duration, VTD duration, age at onset, or HY stage were observed between male and female patients.

**Figure 5:**
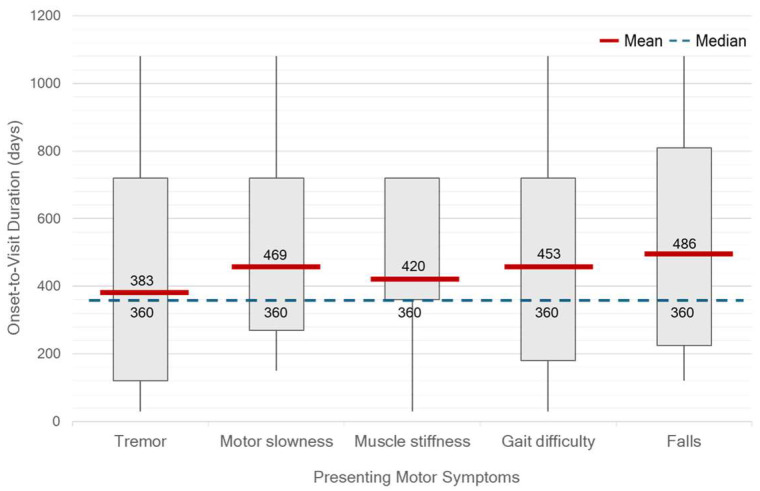
Onset-to-visit (OTV) duration by presenting motor symptoms in 109 Thai patients with Parkinson’s disease. Red lines indicate mean values, blue dashed lines indicate medians, and numbers indicate mean/median. Patients presenting with tremor tended to have shorter OTV durations compared with those presenting with motor slowness, muscle stiffness, gait difficulty, or falls, suggesting that tremor more often prompted earlier medical consultation.

At the time of diagnosis, the median HY stage was 2 (range, 1–4). Only 32.10% of patients were classified as HY stage 1, while 9.17% were diagnosed at advanced stages (HY stage 3–4). Unilateral motor symptom predominance was observed in 93.57% of patients, most commonly on the right side (52.29%). Tremor was the most frequent initial symptom, reported in 75.22% of patients, followed by gait difficulty (38.45%), slowness of movement (37.61%), muscle stiffness (16.51%), and falls (9.17%). No significant differences in OTV duration were observed across different initial motor symptoms. However, patients presenting with tremor tended to seek clinical evaluation earlier than others. (Figure 5.) On neurological examination, bradykinesia was identified in 85.32% of patients, resting tremor in 80.73%, rigidity in 73.39%, and postural instability in 22.02%. A significant discrepancy was found between self-reported slowness of movement and bradykinesia identified during clinical examination (*p* < 0.01).

Among non-motor symptoms, constipation was the most prevalent (32.11%), followed by rapid eye movement sleep behavior disorder (RBD) (23.85%), anxiety (12.84%), insomnia (12.84%), and depression (4.58%). Regarding imaging for diagnosis, approximately one-third of patients (30.28%) did not undergo any neuroimaging studies. Among those who did, brain magnetic resonance imaging (MRI) was performed more frequently (47.71%) than computed tomography (CT) (22.02%). Levodopa was the most initiated treatment following diagnosis, prescribed in 88.07% of patients, followed by dopamine agonists in 11.01%, and no treatment (0.92%).

In 55.96% of patients, initial Parkinsonian features were not recognized as such. The most common alternative diagnoses included tremor disorders (21.10%), orthopedic conditions (17.76%), and ischemic stroke (6.42%) (Table 3). The rate of initially unrecognized parkinsonism was significantly higher among patients first evaluated by non-neurologists compared to neurologists (81.97% vs. 18.03%, *p* < 0.01). Furthermore, patients who initially presented with muscle stiffness (77.78% vs. 51.65%, *p* = 0.04) and gait difficulty (69.05% vs. 47.76%, *p* = 0.03) were more likely to be misdiagnosed.

Correlation coefficients analysis revealed a moderate negative correlation between the OTV duration and years of education (r = −0.37, *p* < 0.01). Weak positive correlations were observed between OTV duration and isolated living arrangement (r = 0.29, *p* = 0.02) and bilateral motor symptom involvement (r = 0.26, *p* = 0.05), HY stage at diagnosis (r = 0.19, *p* = 0.04), and the presence of slowness of movement (r = 0.20, *p* = 0.04). For the VTD duration, a moderate positive correlation was found with initial assessment by non-neurologists (r = 0.38, *p* < 0.01), and a mild positive correlation was noted for the presence of rigidity symptoms (r = 0.22, *p* = 0.02).

## 4. Discussion

The results of this study indicate that the longest delay in the diagnostic process for PD occurs between the onset of motor symptoms and the patient’s decision to seek medical attention. On average, patients waited approximately 12 months before their first medical consultation, underscoring delays in early symptom recognition and potential barriers to accessing healthcare services in Thailand. Compared to earlier Western studies conducted over a decade ago, which reported median durations of 4 months in New York City and 11 months in Cambridge [5,6], the delay observed in our contemporary Thai cohort is notably longer, despite overall improvements in global health literacy and healthcare infrastructure. 

In contrast, the median duration from the first medical visit to diagnosis in our cohort was relatively short. The overall duration was 10 days, and among patients who were initially assessed by non-neurologists, the median duration was 30 days. These intervals are comparable to Western cohorts [5,6]. This efficiency reflects the availability of neurologists at a tertiary hospital but may not be generalizable to settings with limited specialist access.

We found no gender-related differences in time to PD diagnosis, in contrast to previous studies that report delayed diagnosis among women [6]. This finding may reflect comparable healthcare-seeking behaviors and more equitable access to medical services between sexes in Thailand. The predominance of elderly male patients in our cohort aligns with the typical demographic profile of PD reported in both Thai and global populations [5,6,7,8]. 

Beyond sex-related patterns, social context played a critical role in influencing diagnostic timing. Patients with higher educational attainment and those living in extended family settings, common in Thai and other Asian societies, were more likely to seek medical attention earlier, likely because of greater disease awareness and encouragement from family members. In addition, cultural factors also contribute to delays. In Thai society, patients often tolerate mild or non-disabling symptoms and avoid seeking medical care until symptoms become more severe or disruptive. This reluctance may be reinforced by fear of diagnosis, concerns about treatment costs, or the perceived inconvenience of hospital visits. Such health-seeking behaviors likely prolong the OTV duration and delay timely recognition of PD.

Healthcare entitlement also emerged as a key determinant of diagnostic delay. Patients with private insurance experienced a significantly shorter median duration from symptom onset to diagnosis compared to those enrolled in other benefit schemes, particularly the UCS. Although UCS is Thailand’s national insurance program intended to ensure universal access regardless of socioeconomic status, its multi-tiered referral system can create delays before patients reach specialist care. In contrast, patients with financial flexibility or access to streamlined schemes such as the CSMBS can more readily consult neurologists or tertiary care centers, leading to earlier diagnosis. Indeed, CSMBS was the most common healthcare coverage among patients in this cohort, indicating that many received full reimbursement for medical expenses. This facet likely contributed to the relatively shorter diagnostic timelines observed in our study population, compared to what might be expected in the broader Thai population.

Educational level and household income also appear to contribute meaningfully to diagnostic disparities. Lower education levels among members of older generations may partly explain reduced symptom recognition, a notion consistent with the observed correlation between education and diagnostic delay. The household income data, while incomplete, revealed that over half of the patients had a monthly income below 50,000 Thai Baht. These lower-income patients are more likely to be covered under UCS, further compounding diagnostic delays due to structural barriers.

Taken together, these findings illustrate how disparities in education, income, and healthcare coverage intersect to shape the diagnostic journeys of PD patients. Addressing these systemic inequities through public health education, enhanced primary care screening, and more efficient referral mechanisms, particularly within the UCS, will be essential in promoting earlier diagnosis and ensuring more equitable brain health outcomes.

A family history of PD was documented in less than half of the patients, a finding that may be attributable to incomplete medical records or insufficient emphasis on family history during clinical assessment. Nevertheless, the proportion of patients with a confirmed positive family history was consistent with previous reports [5,7]. Although the majority of PD cases are considered sporadic, they are likely influenced by complex interactions between genetic predisposition and environmental factors [9]. Documenting family history may provide valuable insights into hereditary risk and facilitate the identification of genetic variants and inheritance patterns in future research. 

This study found substantial variation in the distance between patients’ residences and the hospital, ranging from being within the same province to being in other provinces and even in different regions. These differences may reflect disparities in healthcare coverage. Patients receiving care under the UCS were generally referred from nearby provincial hospitals, whereas those covered by the CSMBS or who self-paid were more likely to choose their healthcare providers based on the hospital’s reputation and the availability of specialists. These findings suggest that healthcare entitlement plays an important role in determining both referral patterns and access to expert care.

Interestingly, Parkinsonian features were initially unrecognized or misdiagnosed for more than half of the patients with PD, most commonly by non-neurologists during their first medical assessment. Patients who presented with unilateral muscle stiffness and gait difficulty were frequently misdiagnosed as having suffered a stroke or being afflicted with other orthopedic conditions. Initial misdiagnosis not only delays appropriate treatment but may also result in unnecessary medication use, excessive diagnostic investigations, and unwarranted surgical interventions. These findings underscore the urgent need to improve awareness and diagnostic proficiency related to PD among a broader range of healthcare providers. Movement disorder specialists and neurologists played a pivotal role in establishing an accurate diagnosis of PD. Therefore, expanding access to neurologists and movement disorder specialists, along with implementing efficient consultation and referral systems, is crucial for ensuring early and accurate diagnosis of PD.

In terms of motor symptoms, a tremor was the most common presenting feature in this study, a result consistent with previous findings [5,10]. It often served as the main symptom prompting patients to seek medical attention and guiding physicians toward a diagnosis of PD. However, it is important to recognize that tremors are not required for diagnosis, as approximately 15 to 20 percent of patients with PD do not exhibit tremors [10]. Additionally, patients with EOPD associated with gene mutations, such as PRKN (Parkin) mutations, may exhibit slower disease progression, dystonic features involving the lower limbs, and often an absence of tremors. These atypical clinical presentations may contribute to diagnostic delays [11,12].

Notably, a clear discrepancy was observed between self-reported slowness of movement and bradykinesia identified during clinical examination, highlighting the under-recognition of subtle or mild bradykinetic symptoms by patients. In addition, patients with very-late-onset PD appeared to be diagnosed later than those in other age groups. This finding may reflect a tendency to attribute symptoms to the normal aging process rather than a pathological condition. Taken together, these findings emphasize the need to improve both public and clinical awareness of non-tremor motor manifestations of PD. Additionally, approximately half of the patients were diagnosed at a moderate stage, as indicated by HY stage 2, suggesting that the symptoms prompting medical consultation often emerge at a later stage of the disease, typically when bilateral motor involvement has already developed. Therefore, greater emphasis should be placed on the recognition of early-stage symptoms, including those in the prodromal phase. 

In terms of non-motor symptoms (NMSs), the prevalence observed in this study was lower than that previously reported [6], suggesting potential under-recognition or under-documentation of NMSs by physicians. A comprehensive evaluation of NMSs should be an integral part of routine clinical PD assessment. Continuing medical education on the broad spectrum of PD manifestations is essential to improve diagnostic accuracy and optimize patient care.

Regarding the use of neuroimaging in diagnosis, approximately 30% of patients did not undergo any brain imaging studies. This finding is consistent with clinical practice when the presentation is typical and fulfills established diagnostic criteria. In such cases, neuroimaging may be deemed unnecessary, particularly in settings where resources are limited. 

In the context of diagnostic disparities, biomarkers offer a promising avenue for improving early recognition of PD [13]. While traditional imaging biomarkers such as dopamine transporter single-photon emission computed tomography (DAT-SPECT) remain costly and limited in availability, digital biomarkers present a more scalable and accessible alternative in middle-income countries like Thailand. With high smartphone penetration in Thailand [14], nationwide digital health initiatives, such as a “Check PD” campaign, could empower individuals aged 40 and older to self-screen using a smartphone-based application [15]. These platforms, which assess tremor, dexterity, gait, and voice, may help identify subtle abnormalities long before patients seek medical care, reducing reliance on physician expertise and mitigating disparities in under-resourced areas. 

Awareness of prodromal symptoms, including constipation and REM sleep behavior disorder, also remains limited. Education and training for both the public and primary care physicians could improve timely recognition and referral. Locally developed tools, such as the non-motor symptoms questionnaire (NMSQ) application [16], further demonstrate the potential of digital health solutions to increase awareness and patient engagement. While not diagnostic, these approaches can act as diagnostic equalizers, expanding access to early detection strategies and advancing brain health equity.

In this cohort, levodopa was the most frequently initiated treatment, prescribed for almost 90% of patients following diagnosis. This high rate reflects current clinical practices and recommendations, particularly in resource-limited settings where levodopa remains the most effective and accessible therapy for the motor symptoms of PD. Its early and widespread use in this population also suggests that most patients presented with symptoms that were advanced enough to warrant pharmacologic intervention, further underscoring the need for earlier detection and timely referral in order to minimize functional impairment at treatment initiation. 

Despite the apparent familiarity of diagnostic delay issues in PD, the context-specific challenges faced by patients in LMICs like Thailand remain underexplored. Our study highlights how health entitlement schemes, sociocultural dynamics, and systemic referral structures uniquely influence the diagnostic journey in this population. By providing empirical data from a Southeast Asian setting, our study emphasizes the need for localized evidence in order to guide equitable healthcare policies. Addressing diagnostic delays represents a “known but neglected” issue in brain health equity. Reducing delays through public education, primary care training, and streamlined referral systems is essential to ensure that no patient is left behind.

Several limitations should be acknowledged when interpreting the findings of this study. First, the retrospective design may have introduced missing-data and documentation bias, although the use of standardized diagnostic criteria and structured data abstraction enhanced consistency and reliability. Second, as a single-tertiary-care-center study, its generalizability may be limited. However, the hospital serves a wide catchment area with both urban and rural populations, so the findings likely reflect real-world diagnostic challenges in similar settings. Finally, although the sample size was modest, it was drawn from a clearly defined cohort of newly diagnosed PD patients, allowing for robust analysis of diagnostic intervals, symptomatology, and system-level factors. 

## 5. Conclusions

The greatest diagnostic delay in PD arises before the first clinical visit, underscoring the need for improved symptom recognition and healthcare access. Tremor remains the most common presentation, but non-tremor and non-motor symptoms are frequently overlooked, contributing to misdiagnosis and late referral. Neurologists are central to diagnostic accuracy, yet equitable access is hindered by structural barriers, especially within UCS. Policy priorities should include physician training, streamlined referral pathways, and the integration of biomarker-supported digital tools and telemedicine. Together, these measures can promote earlier diagnosis and ensure that no patient is left behind in the pursuit of brain health equity.

## Figures and Tables

**Table 1:** Demographic and clinical characteristics of patients at the time of Parkinson’s disease symptom onset.

Variable	N	Value
Male sex	109	65 (59.63%)
Age at onset (years, mean ± SD)	109	69.24 ± 9.29
<50		4 (3.67%)
50–59		12 (11.01%)
60–69		38 (34.86%)
70–79		34 (31.19%)
≥80		21 (19.27%)
Family history of Parkinson’s Disease	51	6 (11.76%)
Education (years, mean ± SD)	89	12.02 ± 5.24
Distance from hospital (km, median (IQR))	109	38.0 (19.0–69.5)
Living arrangement	89	
Living alone		5 (5.62%)
Living with partner/spouse		18 (20.22%)
Nuclear family		22 (24.72%)
Extended family		44 (49.44%)
Healthcare system	109	
Civil Servant Medical Benefit Scheme (CSMBS)		68 (62.38%)
Universal Coverage Scheme (UCS)		17 (15.59%)
Social Security Scheme (SSS)		5 (4.58%)
Self-pay/private insurance		19 (17.43%)
Family monthly income (Thai Baht)	46	
<10,000		5 (10.87%)
10,000–49,999		20 (43.48%)
50,000–99,999		14 (30.43%)
≥100,000		7 (15.22%)
Primary physician at initial evaluation	109	
Neurologist/neurology resident		20 (18.35%)
Movement disorder specialist		21 (19.27%)
Internal medicine physician/resident		23 (21.10%)
General practitioner		18 (16.51%)
Orthopedic specialist		18 (16.51%)
Others		9 (8.26%)

Abbreviations: SD, standard deviation; IQR, interquartile range.

**Table 2:** Time durations related to Parkinson’s disease diagnosis by the initial evaluating physician.

Duration (Median, IQR, Days)	All (n = 109)	Neurologist (n = 41)	Non-Neurologist (n = 68)	*p*-Value
Onset-to-visit	360 (150–720)	360 (120–630)	360 (157–720)	0.69
Visit-to-diagnosis	10 (1–30)	1 (1–1)	30 (10–68)	<0.01
Onset-to-diagnosis	370 (181–721)	361 (121–631)	390 (330–733)	<0.01

Note: Values are presented as median (interquartile range, IQR) in days.

**Table 3:** Initial diagnoses given at first clinical evaluation among patients subsequently diagnosed with Parkinson’s disease.

Initial Diagnosis	Number (%)
Parkinson’s disease/parkinsonism	48 (44.04)
Tremor disorders: essential tremor, physiological tremor	23 (21.10)
Orthopedic conditions: cervical/lumbar spondylosis, shoulder stiffness, osteoarthritis	19 (17.76)
Cerebrovascular diseases: ischemic stroke, transient ischemic attack	7 (6.42)
Mood and mental disorders: anxiety, cognitive decline, depression	6 (5.50)
GI symptoms: dyspepsia, constipation, bloating	4 (3.67)
Others: insomnia, aching pain	2 (1.83)

## Data Availability

The datasets generated during the current study are available from the corresponding author on reasonable request.

## Abbreviations

The following abbreviations are used in this manuscript:

CT	Computed Tomography
CSMBS	Civil Servant Medical Benefit Scheme
DAT-SPECT	Dopamine Transporter Single-Photon Emission Computed Tomography
EOPD	Early-Onset Parkinson’s Disease
HY	Hoehn and Yahr
IQR	Interquartile Range
LMICs	Low- and Middle-Income Countries
MDS-PD	Movement Disorder Society Parkinson’s Disease diagnostic criteria
MRI	Magnetic Resonance Imaging
NMS	Non-Motor Symptoms
NMSQ	Non-Motor Symptoms Questionnaire
OTD	Onset-to-Diagnosis
OTV	Onset-to-Visit
PD	Parkinson’s disease
PRKN	Parkin gene
RBD	Rapid Eye Movement Sleep Behavior Disorder
SD	Standard Deviation
UCS	Universal Coverage Scheme
VTD	Visit-to-Diagnosis
